# Efficacy and safety of anticoagulant prophylaxis for prevention of postoperative venous thromboembolism in Japanese patients undergoing laparoscopic colorectal cancer surgery

**DOI:** 10.1002/ags3.12279

**Published:** 2019-07-22

**Authors:** Taishi Hata, Masayoshi Yasui, Masataka Ikeda, Masakazu Miyake, Yoshihito Ide, Masaki Okuyama, Masakazu Ikenaga, Kotaro Kitani, Shunji Morita, Chu Matsuda, Tsunekazu Mizushima, Hirofumi Yamamoto, Kohei Murata, Mitsugu Sekimoto, Riichiro Nezu, Masaki Mori, Yuichiro Doki, For Clinical Study Group of Osaka University Colorectal Group (CSGOCG) Investigators

**Affiliations:** ^1^ Department of Gastroenterological Surgery Graduate School of Medicine Osaka University Suita-city, Osaka Japan; ^2^ Department of Gastroenterological Surgery Osaka International Cancer Institute Osaka-city, Osaka Japan; ^3^ Department of Surgery Hyogo College of Medicine Nishinomiya-city, Hyogo Japan; ^4^ Department of Surgery Osaka General Medical Center Osaka-city, Osaka Japan; ^5^ Department of Surgery Yao Municipal Hospital Yao-city, Osaka Japan; ^6^ Department of surgery Kaizuka City Hospital Kaizuka-city, Osaka Japan; ^7^ Department of Gastroenterological surgery Higashiosaka City Medical Center Higashiosaka-city, Osaka Japan; ^8^ Department of Gastroenterological Surgery Kindai University Nara Hospital Ikoma-city, Nara Japan; ^9^ Department of Gastroenterological Surgery Toyonaka Municipal Hospital Toyonaka-city,Osaka Japan; ^10^ Department of Therapeutics for Inflammatory Bowel Diseases Graduate School of Medicine Osaka University Suita-city, Osaka Japan; ^11^ Department of Molecular Pathology Division of Health Sciences Graduate School of Medicine Osaka University Suita-city, Osaka Japan; ^12^ Department of Surgery Kansai Rosai Hospital Amagasaki-city, Hyogo Japan; ^13^ Department of surgery Nishinomiya Municipal Central Hospital Nishinomiya-city, Hyogo Japan; ^14^ Department of Surgery and Science Graduate School of Medical Sciences Kyushu University Fukuoka-city, Fukuoka Japan

**Keywords:** anticoagulant prophylaxis, colorectal cancer, laparoscopic surgery, venous thromboembolism

## Abstract

**Aim:**

To investigate the efficacy and safety of anticoagulant prophylaxis to prevent postoperative venous thromboembolism (VTE) during laparoscopic colorectal cancer (CRC) surgery, which is unknown in Japanese patients.

**Methods:**

We conducted this randomized controlled trial at nine institutions in Japan from 2011 to 2015. It included 302 eligible patients aged 20 years or older who underwent elective laparoscopic surgery for CRC. Patients were randomly assigned to an intermittent pneumatic compression (IPC) therapy group or to an IPC + anticoagulation therapy group. Anticoagulation therapy comprised fondaparinux or enoxaparin for postoperative VTE prophylaxis. Postoperative VTE was diagnosed based on enhanced multi‐detector helical computed tomography. The primary endpoint was VTE incidence, including asymptomatic cases, the secondary endpoint was incidence of major bleeding, and we conducted an intention‐to‐treat analysis. This study is registered in UMINCTR (UMIN000008435).

**Results:**

Postoperative VTE incidence was 5.10% with IPC therapy (n = 157) and 2.76% with IPC + anticoagulant therapy (n = 145; *P *=* *.293). We identified no symptomatic VTE cases. The major bleeding rates were 1.27% with IPC alone and 1.38% with the combination (*P *=* *.936). The overall bleeding rates were 7.69% for enoxaparin and 13.6% for fondaparinux (*P *=* *.500), and there were no bleeding‐related deaths.

**Conclusion:**

Anticoagulant prophylaxis did not reduce the incidence of VTE and the incidence of major bleeding was comparable between the two groups. Usefulness of perioperative anticoagulation was not demonstrated in this study. Pharmacological prophylaxis must be restricted in Japanese patients with higher risk of VTE.

## 1 INTRODUCTION

Venous thromboembolism (VTE) is a common surgical complication. The incidence of fatal VTE ranges from 0.1% to 0.8%,^1^ and VTE incidence in Japan is almost the same as in Western countries. In Japan, the age‐adjusted mortality rate with VTE increased from 1951 to 2000,^2^ and the condition arises in 24.3% of abdominal surgery patients, including in asymptomatic cases.^3^

VTE occurs in up to 20% of cancer patients and is a leading cause of death in this patient population.^4^, ^5^ The risk of VTE differs according to cancer subgroup, treatment, and procedure,^6^ with the highest risk during the initial period after a diagnosis of malignancy.^7^ Thus, VTE can be considered a potentially fatal but preventable complication after major cancer surgery,^8^ and prophylaxis is crucial in this setting. Of note, some of the mechanisms that give rise to cancer also can lead to VTE. For example, cancer cells can directly promote blood coagulation by generating thrombin or indirectly promote it by stimulating endothelial cells and circulating mononuclear cells to synthesize and express procoagulant factors.^9^, ^10^, ^11^

Since the early 1990s, laparoscopic surgery has revolutionized the field of gastrointestinal surgery,^12^ and this approach for major cancer surgery has become increasingly common. However, uniform guidelines are lacking on the use of anticoagulant prophylaxis, with little available evidence to justify its routine use in laparoscopic cancer surgery.^13^, ^14^

A search of PubMed, PubMed Central, and Google Scholar for the terms “colon or colorectal surgery” and “VTE” and of reference lists of retrieved articles identified 20 relevant papers.^15^, ^16^, ^17^, ^18^, ^19^, ^20^, ^21^, ^22^, ^23^, ^24^, ^25^, ^26^, ^27^, ^28^, ^29^, ^30^, ^31^, ^32^ Many of these studies were retrospective analyses that relied on database searches. They showed that VTE rates are generally lower in patients undergoing laparoscopic compared to open surgery. Of these identified studies, six had a prospective cohort design, two of which involved laparoscopic surgery. Only one study was a randomized trial,^28^ which compared the effectiveness and safety of low‐dose heparin versus low‐molecular‐weight heparin (enoxaparin) as VTE prophylaxis after colorectal surgery. In that study, VTE rates were the same in both groups, without bleeding complications or deaths from pulmonary embolism (PE), suggesting the safety and effectiveness of both anticoagulants. However, the need for anticoagulant prophylaxis to prevent VTE after laparoscopic colorectal cancer (CRC) surgery among patients of Asian descent, including Japanese patients, is unknown.

For this reason, we conducted this randomized study to investigate the clinical need for anticoagulant prophylaxis to prevent postoperative VTE in patients who undergo laparoscopic CRC surgery.

## 2 METHODS

### 2.1 Study design and participants

We conducted this multicenter, open‐label, phase III randomized controlled trial at nine institutions in Japan from October 1, 2011, to December 31, 2015. The study was organized by the Clinical Study Group of the Osaka University Colorectal Group (CSGOCG), which consists of hospitals affiliated with the Department of Gastroenterological Surgery, Graduate School of Medicine, Osaka University. The study protocol was registered on the website of the University Hospital Medical Information Network, Japan (protocol ID: UMIN000008435).

Patients who were undergoing laparoscopic colorectal surgery who had an additional risk factor for VTE were included. As noted in the Japanese VTE guidelines, these additional risk factors include “thrombotic disorder, history of VTE, malignant disease, cancer chemotherapy, serious infection, central venous catheterization, long‐term bed rest (more than 24 hours after surgery), leg paralysis, leg cast fixation, hormone therapy, obesity (body mass index 25 kg/m^2^ or more), and varicose veins of the lower extremities.” Other inclusion criteria were as follows: confirmed CRC by endoscopic examination; age ≥20 years or older; sufficient organ function, per laboratory data showing white blood cell count ≥3000/mm^3^, platelets ≥100 000/mm^3^, total bilirubin ≤2.0 mg/dL, liver enzymes ≤100 IU/L, and serum creatinine ≤1.5 mg/dL; pre‐operative d‐dimer <1 μg/mL or less than twice the institution limit for excluding asymptomatic deep vein thrombosis (DVT); symptomatic DVT; and provision of written informed consent.

The exclusion criteria were as follows: active bleeding or with thrombocytopenia (platelets <10 × 10^4^/μL); risk of bleeding, including gastrointestinal ulcers, diverticulitis, colitis, acute bacterial endocarditis, uncontrolled severe hypertension, or uncontrolled diabetes mellitus; severe liver dysfunction (Child C); known hypersensitivity to unfractionated heparin, low‐molecular‐weight heparin, or heparinoids; history of intracranial bleeding; having undergone central cranial surgery, spine surgery, or ophthalmic surgery within 3 months before registration in the study; severe renal dysfunction (creatine clearance <20 mL/min); known hypersensitivity to contrast media; or any condition that made the patient unfit for the study, as determined by the attending physician.

The study was conducted in accordance with the ethics principles set forth in the Declaration of Helsinki, and the institutional review boards at each hospital approved the study protocol. All patients provided written informed consent before randomization. We did not collect data on the number of patients who were approached and assessed for eligibility.

### 2.2 Randomization and masking

Investigators registered the patients in the study, and treatment allocation was performed preoperatively after study eligibility criteria were confirmed. Patients were randomly assigned (1:1) to either the intermittent pneumatic compression (IPC) therapy group or to the IPC + anticoagulation therapy group, using permuted blocks of four stratified by institution, gender, age, and cancer location (colon or rectum). The surgeon was informed of the patient's treatment allocation and performed the procedures. Patients and investigators were not masked regarding group assignment. The data center, which was based at the Multicenter Clinical Study Group at Osaka University, was responsible for treatment allocation, central monitoring, and statistical analyses under the supervision of the study statistician.

### 2.3 VTE prophylaxis

All patients wore graduated compression stockings and received IPC. In the IPC therapy group, the attending physician used compression stockings and IPC without anticoagulant therapy for VTE prophylaxis. In the IPC + anticoagulation therapy group, the physician used compression stockings and IPC plus anticoagulant therapy. Either fondaparinux (Arixtra®; GlaxoSmithKline) or enoxaparin (Kurekisan®; Kaken Pharmaceutical Co., Ltd.) was used. The surgeon made the choice of anticoagulant. Unfractionated heparin is also recommended in the Japanese guidelines, but enoxaparin and fondaparinux only were used in this study.^33^

Administration of fondaparinux or enoxaparin began 24 ± 2 hours after surgery, once hemostasis was established, following the Japanese regimen for VTE prevention. Fondaparinux (2.5 mg) was given once daily for 4‐8 days, and enoxaparin (20 000 IU) was given twice daily for 7‐14 days. The day of surgery was defined as day 1. The study protocol included the approved use of epidural anesthesia as necessary. The catheter had to be removed at least 2 hours before starting the anticoagulant. The primary endpoint was the incidence of VTE, and the secondary endpoint was the incidence of major bleeding.

### 2.4 Assessment and outcome definitions

#### 2.4.1 Diagnosis of VTE

If clinically suspicious VTE symptoms were noted, such as dyspnea, chest pain, or decreased percutaneous arterial oxygen saturation (SpO_2_), we performed enhanced multi‐detector helical computed tomography (MDCT) with contrast media, pulmonary scintigraphy, or pulmonary arteriography to immediately diagnose PE. If lower extremity swelling occurred, we performed ultrasonography, MDCT, or ascending phlebography to diagnose DVT.

If VTE was not suspected, the IPC therapy group underwent ≥8‐channel MDCT on postoperative days 7‐16. In the IPC + anticoagulation therapy group, MDCT was performed after anticoagulant therapy ended on postoperative days 7‐16. To diagnose VTE, sections of 0.5‐0.625 mm were acquired from the chest, body, and legs. A total of 300 mg/mL (maximum of 150 mL) of iodinated contrast medium was injected into the intravenous catheter. The injection rate was 3.0 mL/s. A radiologist interpreted all multislice computed tomography pulmonary angiography scans. SpO_2_, plasma d‐dimer, platelet count, and liver function were prospectively recorded preoperatively and on postoperative days 1, 3, and 7. The radiologist interpreted the CT scans without any identifying information about the patients.

#### 2.4.2 Classification of major and minor bleeding

Bleeding was classified as major if it met ≥1 of the following conditions: fatal bleeding; retroperitoneal or intracranial bleeding; bleeding of critical organs (intraocular, adrenal, endocardial, or spinal bleeding); surgical site bleeding that required surgical intervention; or clinically overt bleeding with a decrease in hemoglobin of ≥2 g/dL, or the need for transfusion of ≥800 mL of red blood cells or whole blood within 48 hours from suspicion to bleeding symptoms. Minor bleeding was defined as bleeding that did not meet any of the major bleeding criteria.

### 2.5 Statistical analysis

We planned a sample size of 150 patients per treatment group when we designed the trial. The sample size was calculated using the following assumptions to 80% power with a two‐sided significance level of 0.05 to detect superiority in reduced VTE frequency.

In earlier studies, the frequency of VTE was 10.8% with fondaparinux prophylaxis and 17.6% with IPC in patients who underwent abdominal surgery.^14^ In addition, VTE incidence was 1.2% in the enoxaparin group and 19.4% in the IPC group.^34^ Using these data, we estimated that VTE could be anticipated to occur in 17% of patients with IPC therapy and 5% with IPC + anticoagulation therapy, allowing for a loss to follow‐up of roughly 20%.

The analysis was performed on an intention‐to‐treat basis, using JMP Pro 13.1.0 software (SAS Institute Inc., Cary, NC. USA). To evaluate each parameter, the Chi‐squared test or Fisher's exact test was used for categorical data, and Student's *t* test was used for continuous variables. The limit for statistical significance was set at *P *<* *.05.

## 3 RESULTS

### 3.1 Patient eligibility

**Figure 1:**
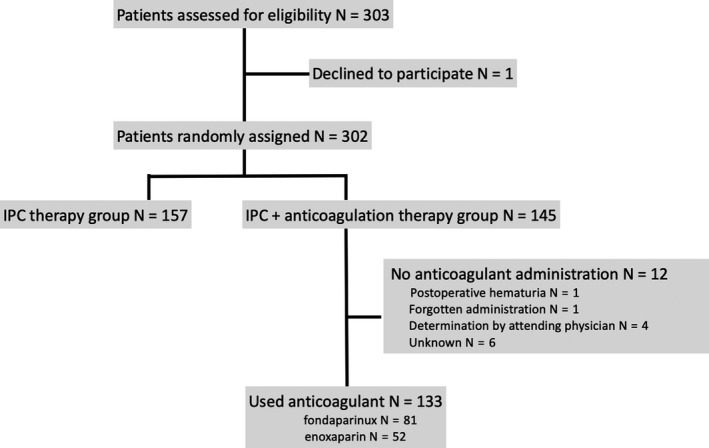
Consolidated Standards of Reporting Trials (CONSORT) flowchart of the study

Figure 1 shows the Consolidated Standards of Reporting Trials (CONSORT) flow diagram for the study, which registered 303 patients. One patient declined to participate after registration. The remaining 302 patients were randomly assigned to the IPC therapy group (n = 157) or the IPC + anticoagulation therapy group (n = 145). In the 145 patients in the latter group, anticoagulation therapy was fondaparinux for 81 and enoxaparin for 52. Another 12 patients in this group did not receive anticoagulation therapy because of postoperative hematuria (n = 1), forgotten administration (n = 1), determination by the attending physician (n = 4), or unknown reasons (n = 6). Table 1 shows the baseline clinical characteristics of the two groups, which were similar.

**Table 1:** Baseline characteristics of the patients in the study

Characteristic	IPC therapy group (n = 157)	IPC + anticoagulation therapy group (n = 145)	*P* value
Male/female	84:72; 54%:46%	84:61; 58%:42%	.476
Mean age	64.9 ± 10.3	65.4 ± 9.5	.691
Weight	58.3 ± 9.75	58.7 ± 11.4	.731
BMI	22.5 ± 3.21	22.6 ± 3.60	.848
Cancer location (colon/rectum)	130/27	130/15	.09
Stage (adenoma/0/I/II/III/IV/unknown)	1/2/57/39/52/0/6	0/5/44/36/49/3/8	.322
Risk factor for VTE (%)			
Diabetes mellitus	9.8	11.5	.700
Past VTE	0.6	0	1.000
Chronic renal failure	1.3	1.4	1.000
Past history of stroke	1.3	0.7	1.000
Past history of angina	3.2	2.1	.725
Past history of cardiac infarction	0	0	—
Varicose veins	3.2	4.1	.763
Dyslipidemia	15.3	16.6	.875
Leg paralysis	0	1.4	.230
Laboratory data			
PreOP d‐dimer (μg/mL)	0.55 ± 0.40	0.53 ± 0.55	.710
APTT (s)	30.1 ± 3.66	30.2 ± 3.26	.726
Hemoglobin (g/L)	13.3 ± 0.60	14.3 ± 0.60	.216
Platelets (×10^4^/L)	33.0 ± 3.41	32.3 ± 3.54	.872
Surgical data			
Open conversion rate	1.9%	4.8%	.153
OP times	297 ± 11.3	280 ± 311.8	.309
Blood loss (mL)	84.7 ± 17.6	97.9 ± 18.3	.606
VTE prophylaxis			
IPC duration (d)	1.19 ± 0.58	1.31 ± 0.96	.272
Anticoagulant duration (d)	—	6.0 ± 2.05	

Abbreviations: APTT, activated partial thromboplastin time; BMI, body mass index; IPC, intermittent pneumatic compression; VTE, venous thromboembolism.

### 3.2 Evaluation of VTE

The VTE incidence was 5.10% with IPC therapy and 2.76% with IPC + anticoagulation (*P *=* *.382). The incidence of PE was 1.91% with IPC and 0.69% with the combination (*P *=* *.623); PE + proximal DVT occurred in 3.82% and 2.76% (*P *=* *.752), respectively; and distal DVT arose in 1.34% and 0%, respectively (Table 2). The groups did not differ statistically from each other, and symptomatic VTE did not occur in this study.

**Table 2:** Incidence of VTE in the patients in this study

	VTE (−)	VTE (+)	Location (n)	Frequency (%)		*P*
IPC therapy group	149	8	PE (3)	1.91	5.10%	.382
			Pulmonary artery (1)			
			Pulmonary artery + posterior tibial vein (2)			
			Proximal VTE (3)	2.01		
			External iliac vein/popliteal vein (1)			
			External iliac vein (1)			
			Superficial femoral vein + deep femoral vein (1)			
			Distal VTE (2)	1.34		
			Soleal vein (2)			
IPC + anticoagulation therapy group	141	4	PE (1)	0.69	2.76%	
			Pulmonary artery (1)			
			Proximal VTE (3)	2.13		
			Deep femoral vein (1)			
			Popliteal vein (2)			
			Distal VTE (0)	0		

Abbreviations: IPC, intermittent pneumatic compression; PE, pulmonary embolism; VTE, venous thromboembolism.

### 3.3 Safety outcomes

The incidence of all bleeding events was 5/157 (3.18%) for the IPC therapy group and 19/145 (13.1%) for the IPC + anticoagulation therapy group, with significant differences between groups (*P *=* *.002). There were no deaths related to bleeding, and major bleeding occurred in two patients in each group (*P *=* *.936; Table 3).

**Table 3:** Incidence of bleeding in the patients in this study

	IPC therapy group (%)	IPC + anticoagulation therapy group (%)	*P*
All bleeding	5/157 (3.18)	19/145 (13.1)	.002
Major bleeding	2/157 (1.27)	2/145 (1.38)	.936
Minor bleeding	3/157 (1.91)	17/145 (11.7)	.001

Abbreviation: IPC, intermittent pneumatic compression.

Regarding anticoagulant safety, the incidence of all bleeding was 11/81 (13.6%) with fondaparinux and 4/52 (7.69%) with enoxaparin; thus, enoxaparin had fewer postoperative bleeding events than fondaparinux, but there was no statistical difference in each group. (*P *=* *.5; Table 4). Most of the major bleeding was anastomotic (75%), and minor bleeding was mainly the result of melena (50%) and subcutaneous (36%) bleeding (Table 5).

**Table 4:** Incidence of bleeding in patients in this study who were treated with fondaparinux vs enoxaparin

	Fondaparinux (%)	Enoxaparin (%)	*P*
All bleeding	11/81 (13.6)	4/52 (7.69)	.500
Major bleeding	0/81 (0)	1/522 (1.92)	.386
Minor bleeding	11/81 (13.6)	3/52 (5.77)	.296

**Table 5:** Location of bleeding

	IPC therapy group	IPC + anticoagulation therapy group		
		Fondaparinux	Enoxaparin	Without administration
Major bleeding	Anastomosis 1		Anastomosis 1	Anastomosis 1
	Intrapelvic 1			
Minor bleeding	Melena 2	Melena 3	Melena 1	Melena 1
	Bloody drain discharge 1	Bloody drain discharge 2		Hematuria 1
		Subcutaneous 5	Subcutaneous 1	
		Unknown 1	Unknown 1	

Abbreviation: IPC, intermittent pneumatic compression.

## 4 DISCUSSION

In this study, the incidence of VTE was 5.10% in the IPC therapy group and 2.76% in the IPC + anticoagulation therapy group, with no significant differences between them (*P *=* *.382); however, the VTE incidence rate was lower with anticoagulant use. A more detailed analysis showed that PE incidence was 2.01% and 0.69% (*P *=* *.623) without and with anticoagulant, respectively, and the incidence of PE + proximal DVT was 3.82% and 2.76% (*P *=* *.752), respectively. These findings showed a trend toward lower incidence with use of an anticoagulant but did not unequivocally confirm the usefulness of anticoagulants.

The incidence of DVT as detected by MDCT was much lower than expected. Sakon et al reported in patients who underwent abdominal surgery without active prophylaxis that the incidence of distal and proximal DVT was 20.8% and 2.9%, respectively, as detected by contrast venography.^4^

Other studies have also reported distal DVT incidence of 1.2% with enoxaparin (low‐molecular‐weight heparin) and 19.4% with IPC using venography.^34^ Sugimachi et al^35^ reported frequencies of proximal and distal DVT with elastic stocking and IPC prophylaxis of 1.5% and 9.8%, respectively, as determined using duplex scan after laparoscopic gastrointestinal surgery. The incidence of proximal DVT in the present study was comparable to that reported previously for Japanese patients, but the incidence of distal DVT was much lower. This distinction suggests that the detection rate for distal DVT with MDCT might be lower than with venography or duplex scan.^36^, ^37^, ^38^

The addition of anticoagulation therapy reduced VTE incidence although not significantly, perhaps because of the low incidence of distal DVT or because of the relatively low number of patients recruited. Perioperative anticoagulant prophylaxis with laparoscopic surgery should be carefully considered in patients of Asian descent with CRC.

The Seventh American College of Chest Physicians (ACCP) guidelines consider patients with a proximal DVT risk of 4%‐8% without prophylaxis to be at high risk and needing anticoagulation.^39^ In the present study, despite using IPC and IPC + anticoagulant therapy, the incidence of PE + proximal DVT was 3.82% and 2.76% with each, respectively. With no prevention, the incidence would be expected to be still higher. Thus, according to the ACCP guidelines, the risk of PE + proximal DVT is estimated to be high or greater in these patients. Our results suggested that preventative IPC or IPC + anticoagulant prophylaxis is essential for this patient population. Of note, anticoagulation therapy did not significantly decrease VTE frequency, and anticoagulant prophylaxis may be more appropriate for patients with a high risk of VTE and low bleeding risk.

In this study, there were no bleeding‐related deaths, and the incidence of major bleeding was 1.27% and 1.38% without and with anticoagulants, respectively (*P *=* *.936). Yamaoka et al^40^ reported a major bleeding incidence of 0.6% (2/362) with fondaparinux and 0.8% (5/591) with IPC among colon cancer patients (*P *=* *.715). We previously reported that the incidence of major bleeding in colon cancer patients with anticoagulant prophylaxis using fondaparinux was 0.81% (5/619; 95% CI 0.3%‐1.9%). In that study, there were no bleeding‐related deaths or deaths from other causes during the treatment period.^15^ The major bleeding rate was similar in these reports, including the current work, and the incidence of clinically problematic bleeding was very low. These findings suggest that VTE prophylaxis using any anticoagulant can be safe with appropriate patient selection.

In the current study, we investigated VTE risk factor‐related data in detail to determine their relationship with VTE. However, the number of primary outcome events was lower than expected, and fewer than expected patients had risk factors. Thus, we could not stratify patients according to higher prevalence of VTE.

The study has some limitations. First, the incidence of VTE varies with ethnicity, but this study included only patients of Japanese ancestry. Second, the number of primary outcome events was lower than expected. Finally, the duration of anticoagulation therapy differs in Japan compared to Western countries.

## 5 CONCLUSIONS

Anticoagulant prophylaxis did not reduce the incidence of VTE and the incidence of major bleeding was comparable between the two groups. Usefulness of perioperative anticoagulation was not demonstrated in this study. Pharmacological prophylaxis must be restricted in Japanese patients with higher risk of VTE.

Author Contributions: Authors make substantial contributions to conception and design, and/or acquisition of data, and/or analysis and interpretation of data: TH; Authors participate in drafting the article or revising it critically for important intellectual content: TH, MY, MIE, MMI, YI, MO, MIEN, KK, SM, CM, TM, HY, KM, MS, RN, MM, YD; Authors give final approval of the version to be published: TH, MY, MM, YI, MO, MI, KK, SM, CM, TM, HY, KM, MS, RN, MMO, YD.

## DISCLOSURE

Conflict of Interest: The authors declare they have no conflict of interest.

Funding: This study was not grant funded.

## ETHICS APPROVAL

All procedures performed in studies involving human participants were in accordance with the ethics standards of the institutional and/or national research committee and with the 1964 Helsinki Declaration and its later amendments or comparable ethical standards.

## INFORMED CONSENT

Informed consent was obtained from all individual participants included in the study.
